# Metagenomic next-generation sequencing of osteoarticular tissue for the diagnosis of suspected osteoarticular tuberculosis

**DOI:** 10.1128/spectrum.03598-23

**Published:** 2024-11-08

**Authors:** Guangxuan Yan, Zhifeng Liu, Tianlu Teng, Weijie Dong, Tinglong Lan, Jun Fan, Kai Tang, Shibing Qin, Wenjuan Nie

**Affiliations:** 1 Orthopedics Department, Beijing Chest Hospital affiliated to Capital Medical University, Beijing Tuberculosis & Thoracic Tumor Research Institute, Beijing, China; 2 Beijing Emercency Mecial Center, Beijing, China; 3 Respiratory Department, Beijing Chest Hospital affiliated to Capital Medical University, Beijing, China; 4 Tuberculosis Department, Beijing Chest Hospital affiliated to Capital Medical University, Beijing Tuberculosis & Thoracic Tumor Research Institute, Beijing, China; Icahn School of Medicine at Mount Sinai, New York, New York, USA

**Keywords:** osteoarticular, tuberculosis, metagenomic next-generation sequencing, sensitivity, specificity

## Abstract

The objective of the study is to determine the accuracy of metagenomic next-generation sequencing (mNGS) in diagnosing osteoarticular tuberculosis (TB) infection and to compare it with mycobacteria growth indicator tube (MGIT) and Xpert assays. We retrospectively analyzed 162 patients admitted with suspected osteoarticular TB. Osteoarticular tissue (66.67%) and abscess specimens (33.33%) from patients were tested for MGIT, GeneXpert/RIF, and mNGS. mNGS assay detected 76 cases (46.9%) with bacterial, 63 cases (38.9%) with mycobacterial, 22 cases (13.6%) with fungal, and 1 case (0.6%) with actinomycetal organisms. These 162 pathogens were classified into 21 species. The most frequent species detected was *Mycobacterium tuberculosis complex* (29.0%), followed by *Staphylococcus aureus* (20.4%), *Mycobacterium abscessus* (5.6%), and *Candida albicans* (5.6%). Taking the “gold standard” TB diagnosis as the standard, the positive predictive values of mNGS, Xpert, and MGIT culture were both 100.00%. The negative predictive values of mNGS, Xpert, and MGIT culture and assays were 94.26%, 98.29%, and 88.46%, respectively. The sensitivity of mNGS detection (85.11%) was similar to that of Xpert (95.74%) and higher than that of MGIT culture (68.08%). The specificities of mNGS detection, Xpert, and MGIT culture were both 100.00%. The area under the curve value of the mNGS assay was 0.895 (95% CI: 0.830, 0.960), which was greater than that of the MGIT culture-based assay of 0.840 (95% CI: 0.757, 0.924), which was similar to 0.979 (95% CI: 0.945, 1.000) for Xpert assay. The pathogen detection rate of mNGS in diagnosing suspected osteoarticular TB exceeded that of conventional methods.

**IMPORTANCE:**

In the detection of unknown infectious disease pathogens, the overall efficacy of traditional detection methods, such as culture, is low, and traditional PCR testing is also limited to the gene sequences of known pathogenic microorganisms. Metagenomic next-generation sequencing (mNGS) performs DNA sequencing by studying the entire microbial community genome in a given sample, without the need for isolation and culture. Previous studies have shown that mNGS performs better on pulmonary and extrapulmonary samples when compared with Xpert, traditional pathogenetic tests, and even parallel diagnostics. However, it should be emphasized that only a few studies have explored the performance of mNGS in detecting *Mycobacterium tuberculosis* in clinical samples associated with bone and joint infections. We conducted this retrospective study to provide additional data to support the use of mNGS in the clinical setting to identify pathogens within abscesses or tissue samples associated with bone and joint infections.

## INTRODUCTION

High incidence rates of bone and joint (osteoarticular) infections are observed in human populations worldwide (1). Despite continual technological advancements in medicine, persistent limited bone and joint function in a large proportion of patients with such infections leads to long-term disability and poor life quality, particularly for people infected with *Mycobacterium tuberculosis* (TB) (2). At present, in China, patients with osteoarticular TB account for approximately 40% of patients with extra-pulmonary TB, compared with 15% in developed countries (3). Importantly, osteoarticular TB patients with additional illnesses, especially comorbid immunodeficiencies, frequently experience greater rates of mortality and/or long-term disability than healthier patients (4). Therefore, early and accurate identification of is crucial for achieving significant chemotherapy outcomes and minimizing toxic side effects associated with empirical treatment strategies.

Abscess formation, the host default response to bacterial invasion, is a host pathogen-containment process that walls off invaders to prevent pathogen spread while host immune system cells (comprised mainly of neutrophils) enter the abscess, kill the pathogens, and then die. Consequently, abscesses contain accumulated dead neutrophils and masses of dead and dying bacteria (5) and thus can serve as test specimens to identify bone and joint infection-associated pathogens. However, due to low viability of intra-abscess bacteria, culture-based testing cannot be reliably used to detect only a few surviving bacteria within such specimens and thus cannot be used to rule out specific causes of infection (6). Nevertheless, molecular tests can detect dead pathogenic organisms and thus may be suitable for detecting pathogens in abscess specimens.

Metagenomic next-generation sequencing (mNGS) platforms now permit rapid pathogen detection and species identification from a single clinical sample. It can overcome the limitations of current diagnostic tests, thus allowing for hypothesis-free, culture-independent pathogen detection directly from clinical specimens. In addition to its ability to detect nonviable microorganisms, mNGS testing has a relatively short turnaround time that enables rapid identification of pathogens to support early initiation of targeted treatments. Moreover, mNGS can detect multiple types of clinical samples, such as blood, bronchoalveolar lavage, and cerebrospinal fluid for pathogens, demonstrating the promise of mNGS as a diagnostic tool for infectious diseases (7). Indeed, due to its demonstrated effectiveness in previous comprehensive microbiological investigations, mNGS is now viewed as a viable option for diagnosing patients with suspected infections of unknown etiology (8). In order to evaluate mNGS performance when used for testing of abscess specimens, this study aimed to determine mNGS accuracy for the diagnosis of osteoarticular infection in fresh abscess specimens obtained from patients with suspected osteoarticular TB. Furthermore, mNGS diagnostic performance was compared with that of the mycobacteria growth indicator tube (MGIT) and GeneXpert/RIF assays for the same set of abscess specimens.

## MATERIALS AND METHODS

### Patient enrollment

We retrospectively analyzed 162 patients diagnosed with suspected osteoarticular TB between January 2019 and July 2022 at Beijing Chest Hospital affiliated to Capital Medical University, with inclusion criteria including (i) fever, (ii) bone and joint pain that can’t be alleviated by rest or analgesics, (iii) magnetic resonance imaging abnormalities, and (iv) past and present TB episodes (9). One abscess specimen from each patient was collected for surgical biopsy. And pathogen culture-based MGIT and GeneXpert/RIF assay were conducted for clinical laboratory tests of the samples.

### Diagnostic classification

We used MGIT + XPERT as a composite “gold standard” with either test positive being considered a clinically positive TB case. In this case, mNGS would be compared with MGIT + XPERT for assessment of sensitivity and specificity.

### Laboratory examination

The collected abscess specimens were assessed by smear microscopy, bacterial culture of mycobacterium, and GeneXpert MTB/RIF assay. In short, according to the guidelines issued by the National Tuberculosis Control Plan (10), the smear microscopy was performed. For mycobacterium culture, abscess sample (1 mL) was provided with a 15-minute treatment with N-acetyl-L-cysteine and sodium hydroxide and subsequently neutralized with phosphate-buffered saline (PBS) followed by a 15-minute centrifugation at 3,000 × *g*. Next, each pellet was then seeded into MGIT (11). Eventually, in 2 hours, through following the manufacturer’s instructions, the GeneXpert MTB/RIF assay was performed to determine the existence of *M. tuberculosis* (MTB) DNA in the abscess samples. The mNGS assay was utilized to analyze the material left in each specimen.

### Metagenomic next-generation sequencing

#### Nucleic acid extraction

Samples of osteoarticular tissue were collected from patients according to standard procedures. DNA was extracted using the QIAamp DNeasy Blood & Tissue Kit (Qiagen) according to the manufacturer’s protocols. The quantity and quality of DNA were assessed using Qubit (Thermo Fisher Scientific) and NanoDrop (Thermo Fisher Scientific), respectively.

#### Library preparation and sequencing

DNA libraries were prepared using the KAPA Hyper Prep Kit (KAPA Biosystems) according to the manufacturer’s protocols. Cluster generation, template hybridization, isothermal amplification, linearization, and blocking denaturing and hybridization of the sequencing primers were performed according to the workflow specified by the service provider. Agilent 2100 (Agilent Technologies) was used for quality control, and DNA libraries were 75-bp single-end sequenced on an Illumina Next-Seq 550Dx platform (Dinfectome Inc., Nanjing, China; Vision Medicals Center for Infectious Diseases, Guangzhou, China, and Beijing CapitalBio Medical Laboratory, Beijing, China) .

#### Bioinformatics analysis

We use an in-house-developed bioinformatics pipeline for pathogen identification. Briefly, high-quality sequencing data were generated by removing low-quality reads, adapter contamination, and duplicated and short (length < 36 bp) reads using Trimmomatic (12). Human host sequence was identified by mapping to human reference genome (hs37d5) using bowtie2 software (13). Reads that could not be mapped to the human genome were retained and aligned with microorganism genome database for pathogen identification using Burrows-Wheeler Alignment software (version 0.7.10) (14). Our microorganism genome database contained bacteria, fungi, virus, and parasite genomic sequences (download from https://www.ncbi.nlm.nih.gov/).

#### Interpretation and reporting

We used the following criteria for positive results of mNGS: for *Mycobacterium*, *Nocardia*, and *Legionella pneumophila*, the result was considered positive if a species detected by mNGS had a species-specific read number ≥ 1 (15). For bacteria (excluding *Mycobacterium*, *Nocardia*, and *L. pneumophila*), fungi, virus, and parasites, the result was considered positive if a species detected by mNGS had at least three non-overlapping reads (8, 16). Pathogens detected in the negative “no-template” control (NTC) were excluded when the ratio of reads per million (RPM)_sample_/RPM_NTC_ was ≥10 (5).

### Statistical analysis

To show true positives, false positives, positive predictive value (PPV), true negatives, false negatives, negative predictive value (NPV), sensitivity, and speciﬁcity, 2 × 2 contingency tables were produced after expressing continuous variables as mean ± SD. The diagnostic accuracy of the mNGS assay was compared with the MGIT culture and Xpert assays’ corresponding accuracies using the paired McNemar chi-square test. SPSS Statistics 24.0 (IBM, NY, USA) was performed for the above statistical analyses, and a *P* < 0.05 was considered statistically signiﬁcant.

## RESULTS

### Baseline comparison of demographic and clinical characteristics between the TB and non-TB groups

Of the enrolled 162 patients, 49 were diagnosed as TB and 113 as non-TB; the clinical characteristics of patients were shown in Table 1. Of these 162 patients, 102 (62.96%) were male and 60 (37.04%) were female. The mean age was 52.89 years (range 12.0–83.0 years). Samples were divided into two categories, including osteoarticular tissue (108/162, 66.67%) and abscess specimens (54/162, 33.33%). In the non-TB group, there were more males than females (69.91% and 46.94%, respectively).

**TABLE 1:** Clinical characteristics of all enrolled patients (*n* = 162)

Clinical characteristics	TB (*n* = 49)	Non-TB (*n* = 113)
Age, years (mean ± SD)	50.50 ± 15.67	54.62 ± 15.40
Gender, *n* (%)		
Male	23 (46.94)	79 (69.91)
Female	26 (53.06)	34 (30.09)
Sample type, *n* (%)		
Osteoarticular tissue	30 (61.22)	78 (69.03)
Abscess specimens	19 (18.38)	35 (30.97)

### mNGS data of clinical samples

Subsequently, mNGS was performed on samples from 162 patients. Numbers of the raw sequence reads ranged from 3.1 × 10^6^ to 4.9 × 10^7^ reads, with an average of (2.2 ±  0.9) × 10^7^ reads per sample. The sequencing depth of mNGS for pathogens ranges from 1.0×  to 31.2×, with an average of (1.7 ±  3.4) × per specimen (Table 2).

**TABLE 2:** Description of patients included in this study

ID	Gender	Age	Xpert	Culture	mNGS					The gold standard for the final diagnosis combining pathology and clinical diagnosis
					No. of raw reads	Pathogen	No. of reads mapped to reference pathogen sequences	Sequencing depth	Coverage (%)	
1	Female	53	Negative	Negative	17271853	*Staphylococcus aureus*	313399	9.21	91.180	Non-TB
2	Female	53	Negative	Negative	24862932	*S. aureus*	476948	13.89	91.690	Non-TB
3	Female	69	Negative	Negative	22588474	*Micrococcus luteus*	15	1.00	0.210	Non-TB
4	Female	64	Negative	Negative	24504005	*S. aureus*	329	1.00	0.920	Non-TB
5	Female	12	Negative	Negative	30140199	*S. aureus*	22327	2.23	34.070	Non-TB
6	Female	56	Negative	Negative	27377757	*S. aureus*	845	1.04	1.910	Non-TB
7	Female	19	Negative	Negative	12990852	*Candida parapsilosis*	26	1.00	0.080	Non-TB
8	Female	51	Negative	Negative	42304655	*Mycobacterium avium*	29	1.05	0.240	Non-TB
9	Female	53	Negative	Negative	3126447	*Candida albicans*	362268	31.20	91.500	Non-TB
10	Female	62	Negative	Negative	11611733	*S. aureus*	3	1.00	0.004	Non-TB
11	Female	78	Negative	Negative	12239867	*Escherichia coli*	6	1.00	0.040	Non-TB
12	Female	78	Negative	Negative	16844160	*E. coli*	175	1.00	0.500	Non-TB
13	Female	43	Negative	Negative	31994459	*Escherichia fergusonii*	60	1.10	0.220	Non-TB
14	Female	46	Negative	Negative	17955897	*S. aureus*	258	1.00	0.970	Non-TB
15	Female	76	Negative	Negative	15060796	*S. aureus*	1764	1.02	4.090	Non-TB
16	Female	63	Negative	Negative	25425285	*Brucella*	98	1.00	0.190	Non-TB
17	Female	56	Negative	Negative	17066975	*Brucella*	9053	1.10	9.800	Non-TB
18	Female	14	Negative	Negative	7493664	*Stenotrophomonas maltophilia*	914	1.00	0.460	Non-TB
19	Female	59	Negative	Negative	11836726	*E. fergusonii*	140	1.00	0.400	Non-TB
20	Female	45	Negative	Negative	14390697	*Mycobacterium abscessus*	805	1.48	33.250	Non-TB
21	Female	58	Negative	Negative	13035236	*S. aureus*	1630	1.17	12.030	Non-TB
22	Female	70	Negative	Negative	14644846	*C. parapsilosis*	2005	1.04	3.110	Non-TB
23	Female	59	Negative	Negative	25652030	*M. abscessus*	150	1.29	6.580	Non-TB
24	Female	74	Negative	Negative	18129492	*S. maltophilia*	6	1.03	0.253	Non-TB
25	Female	66	Negative	Negative	16598900	*Aspergillus fumigatus*	150	1.36	1.700	Non-TB
26	Male	56	Negative	Negative	24847545	*S. aureus*	201	1.00	0.560	Non-TB
27	Male	54	Negative	Negative	26659790	*Finegoldia magna*	2047	1.04	3.020	Non-TB
28	Male	59	Negative	Negative	32266346	*Brucella*	728	1.40	48.810	Non-TB
29	Male	64	Negative	Negative	16027523	*Brucella*	124	1.06	10.580	Non-TB
30	Male	61	Negative	Negative	9685610	*S. aureus*	9	1.01	0.020	Non-TB
31	Male	50	Negative	Negative	19476975	*S. aureus*	512437	12.65	96.860	Non-TB
32	Male	64	Negative	Negative	31831269	*Parvimonas micra*	13758	1.82	58.270	Non-TB
33	Male	49	Negative	Negative	17072790	*M. tuberculosis complex*	802	1.23	18.370	Non-TB
34	Male	53	Negative	Negative	36849258	*C. albicans*	988	1.00	0.670	Non-TB
35	Male	68	Negative	Negative	12130965	*S. aureus*	1122	1.03	2.630	Non-TB
36	Male	41	Negative	Negative	19703849	*Nocardia asiatica*	9792	1.20	1.040	Non-TB
37	Male	52	Negative	Negative	18871516	*Brucella*	193	1.00	0.220	Non-TB
38	Male	31	Negative	Negative	4688872	*Prevotella pleuritidis*	6210	1.10	23.400	Non-TB
39	Male	39	Negative	Negative	23752755	*C. albicans*	28	1.00	0.058	Non-TB
40	Male	71	Negative	Negative	24742517	*S. aureus*	13310	1.20	19.810	Non-TB
41	Male	56	Negative	Negative	27866383	*Pseudomonas aeruginosa*	10	1.00	0.030	Non-TB
42	Male	63	Negative	Negative	16867630	*P. aeruginosa*	26	1.00	0.020	Non-TB
43	Male	46	Negative	Negative	22064509	*M. avium*	7551	2.37	9.700	Non-TB
44	Male	19	Negative	Negative	24551668	*F. magna*	23	1.02	0.238	Non-TB
45	Male	66	Negative	Negative	18211296	*M. tuberculosis complex*	906	1.29	7.830	Non-TB
46	Male	43	Negative	Negative	32824929	*S. aureus*	2268	1.04	6.400	Non-TB
47	Male	54	Negative	Negative	10296238	*S. aureus*	362	1.06	0.520	Non-TB
48	Male	61	Negative	Negative	20324730	*M. avium*	30	1.04	1.850	Non-TB
49	Male	66	Negative	Negative	32794499	*F. magna *	41317	2.08	69.750	Non-TB
50	Male	52	Negative	Negative	31827046	*S. aureus*	52	1.02	0.110	Non-TB
51	Male	67	Negative	Negative	11746020	*P. micra*	41	1.02	0.140	Non-TB
52	Male	80	Negative	Negative	36869461	*Coxiella burnetii*	11	1.05	0.040	Non-TB
53	Male	58	Negative	Negative	43255740	*S. aureus*	8	1.00	0.010	Non-TB
54	Male	60	Negative	Negative	28444166	*Propionibacterium acnes*	9	1.00	0.020	Non-TB
55	Male	46	Negative	Negative	17874724	*S. aureus*	63	1.00	0.140	Non-TB
56	Male	46	Negative	Negative	23716255	*S. aureus*	40	1.00	0.100	Non-TB
57	Male	72	Negative	Negative	26577497	*M. abscessus*	12	1.00	0.220	Non-TB
58	Male	33	Negative	Negative	42689837	*P. acnes*	15	1.00	0.180	Non-TB
59	Male	51	Negative	Negative	34458835	*S. aureus*	158	1.00	0.340	Non-TB
60	Male	44	Negative	Negative	22527881	*S. aureus*	7	1.00	0.030	Non-TB
61	Male	29	Negative	Negative	19861262	*S. aureus*	3	1.00	0.010	Non-TB
62	Male	63	Negative	Negative	18821738	*S. aureus*	169	1.00	0.340	Non-TB
63	Male	59	Negative	Negative	12708514	*P. acnes*	20	1.01	1.770	Non-TB
64	Male	67	Negative	Negative	23635545	*S. aureus*	77	1.00	0.180	Non-TB
65	Male	34	Negative	Negative	18358558	*S. aureus*	6	1.30	0.010	Non-TB
66	Male	45	Negative	Negative	20944373	*P. acnes*	44	1.00	0.080	Non-TB
67	Male	52	Negative	Negative	33232996	*P. acnes*	1275886	26.10	100.000	Non-TB
68	Male	59	Negative	Negative	15608790	*P. acnes*	1080	1.10	0.890	Non-TB
69	Male	41	Negative	Negative	24879354	*C. albicans*	66	1.20	0.020	Non-TB
70	Male	68	Negative	Negative	27728411	*S. aureus*	3	1.00	0.617	Non-TB
71	Male	69	Negative	Negative	11868939	*Streptococcus oralis*	281	1.00	0.260	Non-TB
72	Male	64	Negative	Negative	27236564	*Brucella*	1524	1.01	0.880	Non-TB
73	Male	63	Negative	Negative	15617238	*C. burnetii*	5860	1.10	10.090	Non-TB
74	Male	79	Negative	Negative	12473603	*M. abscessus*	7	1.00	0.010	Non-TB
75	Male	57	Negative	Negative	13767936	*S. oralis*	64	1.00	0.080	Non-TB
76	Male	55	Negative	Negative	29290519	*Candida tropicalis*	1792	1.49	43.640	Non-TB
77	Male	73	Negative	Negative	15831692	*Brucella*	14	1.03	1.760	Non-TB
78	Male	47	Negative	Negative	27468249	*P. micra*	45	1.00	0.220	Non-TB
79	Male	79	Negative	Negative	25586636	*M. luteus*	175	1.01	1.100	Non-TB
80	Male	64	Negative	Negative	20518550	*A. fumigatus*	177	1.00	0.040	Non-TB
81	Male	60	Negative	Negative	18832452	*P. aeruginosa*	50	1.00	0.050	Non-TB
82	Male	55	Negative	Negative	17152458	*P. acnes*	46	1.00	0.090	Non-TB
83	Male	45	Negative	Negative	49383332	*M. tuberculosis complex*	88	1.10	0.920	Non-TB
84	Male	74	Negative	Negative	20105950	*M. tuberculosis complex*	6	1.00	0.050	Non-TB
85	Male	67	Negative	Negative	11311628	*C. tropicalis*	35	1.00	0.890	Non-TB
86	Male	53	Negative	Negative	10878916	*C. tropicalis*	14	1.05	1.080	Non-TB
87	Male	72	Negative	Negative	7704955	*M. abscessus*	14	1.00	0.010	Non-TB
88	Male	34	Negative	Negative	21459869	*S. maltophilia*	1025	1.20	2.090	Non-TB
89	Male	62	Negative	Negative	20895072	*C. parapsilosis*	96	1.06	1.210	Non-TB
90	Male	25	Negative	Negative	11091482	*M. tuberculosis complex*	2036	1.22	1.310	Non-TB
91	Male	17	Negative	Negative	12793158	*C. albicans*	71	1.03	0.090	Non-TB
92	Female	74	Negative	Negative	21301370	*M. abscessus*	33	1.00	0.030	Non-TB
93	Female	25	Negative	Negative	16697383	*M. avium*	29	1.03	0.040	Non-TB
94	Male	46	Negative	Negative	38463414	*M. avium complex*	194	1.02	1.000	Non-TB
95	Male	35	Negative	Negative	25461230	*C. tropicalis*	2992	1.41	8.810	Non-TB
96	Male	49	Negative	Negative	29074880	*A. fumigatus*	1735	1.00	2.560	Non-TB
97	Male	32	Negative	Negative	15864569	*C. albicans*	55	1.00	0.090	Non-TB
98	Male	72	Negative	Negative	13136221	*S. aureus*	147033	2.10	95.850	Non-TB
99	Female	64	Positive	Positive	21425598	*M. tuberculosis complex*	19445	1.15	23.440	TB
100	Female	51	Negative	Negative	19416651	*S. aureus*	5638	1.49	5.750	Non-TB
101	Female	47	Negative	Negative	19159937	*M. avium*	352	1.05	2.300	Non-TB
102	Female	83	Negative	Negative	14255550	*C. burnetii*	6253	1.22	8.800	Non-TB
103	Female	63	Negative	Negative	14438620	*S. aureus*	41	1.03	0.940	Non-TB
104	Female	42	Negative	Negative	17985642	*C. albicans*	6541	3.01	8.700	Non-TB
105	Female	76	Negative	Negative	24116529	*M. abscessus*	1456	3.24	38.600	Non-TB
106	Female	46	Negative	Negative	27204976	*C. albicans*	451	1.04	6.470	Non-TB
107	Male	47	Negative	Negative	25093071	*P. micra*	4123	2.28	10.940	Non-TB
108	Male	30	Negative	Positive	26889558	*S. aureus*	8456	1.41	10.820	TB
109	Male	65	Negative	Negative	21009061	*C. tropicalis*	522	1.00	4.570	Non-TB
110	Male	64	Negative	Negative	22703267	*S. aureus*	6	1.00	0.020	Non-TB
111	Male	30	Negative	Negative	21979663	*M. abscessus*	455	1.21	12.450	Non-TB
112	Male	71	Negative	Negative	43600670	*S. maltophilia*	51	1.00	0.070	Non-TB
113	Male	67	Negative	Negative	26572342	*A. fumigatus*	845	1.02	1.750	Non-TB
114	Male	59	Negative	Negative	28600127	*M. luteus*	416	1.18	3.420	Non-TB
115	Male	40	Negative	Negative	20439445	*M. avium*	651	1.23	10.360	Non-TB
116	Male	67	Negative	Positive	21611153	*A. fumigatus*	3854	1.28	3.630	TB
117	Female	51	Positive	Negative	25298982	*M. abscessus*	17	1.00	0.650	TB
118	Female	34	Positive	Positive	16122590	*S. aureus*	668	1.11	4.150	TB
119	Male	63	Positive	Positive	22209396	*C. albicans*	7845	3.00	19.230	TB
120	Male	32	Positive	Negative	33145173	*P. pleuritidis*	2	1.00	4.710	TB
121	Male	47	Positive	Negative	19512572	*E. fergusonii*	3562	1.75	6.300	TB
122	Female	58	Negative	Negative	22152058	*M. tuberculosis complex*	2	1.00	0.002	Non-TB
123	Male	13	Negative	Negative	21459869	*M. tuberculosis complex*	1	1.00	0.001	Non-TB
124	Female	76	Positive	Positive	26412073	*M. tuberculosis complex*	1769	1.03	2.380	TB
125	Female	58	Positive	Positive	21101727	*M. tuberculosis complex*	68	1.00	0.020	TB
126	Female	35	Positive	Positive	15434607	*M. tuberculosis complex*	953	1.02	1.270	TB
127	Female	34	Positive	Negative	16696703	*M. tuberculosis complex*	262	1.01	0.350	TB
128	Female	62	Positive	Positive	17569964	*M. tuberculosis complex*	20	1.20	0.020	TB
129	Female	58	Positive	Positive	31597700	*M. tuberculosis complex*	12	1.00	0.030	TB
130	Female	38	Positive	Negative	41911945	*M. tuberculosis complex*	9	1.00	0.020	TB
131	Female	33	Positive	Positive	13718697	*M. tuberculosis complex*	487	1.00	0.390	TB
132	Female	65	Positive	Negative	14388109	*M. tuberculosis complex*	11379	1.05	13.710	TB
133	Female	65	Positive	Positive	28507787	*M. tuberculosis complex*	666	1.02	0.900	TB
134	Female	70	Positive	Positive	38638302	*M. tuberculosis complex*	16	1.00	0.020	TB
135	Female	25	Positive	Positive	24624865	*M. tuberculosis complex*	40	1.00	0.060	TB
136	Female	55	Positive	Positive	26403615	*M. tuberculosis complex*	5	1.00	0.001	TB
137	Female	56	Positive	Positive	18872919	*M. tuberculosis complex*	1656	1.03	2.290	TB
138	Female	32	Positive	Positive	37598129	*M. tuberculosis complex*	338	1.03	0.490	TB
139	Female	60	Positive	Positive	26434933	*M. tuberculosis complex*	3	1.00	0.006	TB
140	Female	63	Positive	Negative	12592741	*M. tuberculosis complex*	4	1.00	0.002	TB
141	Female	38	Positive	Negative	20041416	*M. tuberculosis complex*	616	1.00	0.724	TB
142	Female	28	Positive	Negative	27255462	*M. tuberculosis complex*	19	1.00	0.026	TB
143	Female	44	Positive	Negative	18555668	*M. tuberculosis complex*	2	1.00	0.001	TB
144	Female	65	Positive	Positive	13079264	*M. tuberculosis complex*	5995	1.20	3.690	TB
145	Female	16	Positive	Negative	15678035	*M. tuberculosis complex*	12	1.00	0.001	TB
146	Male	32	Positive	Positive	25161527	*M. tuberculosis complex*	29	1.00	0.030	TB
147	Male	54	Positive	Positive	23310055	*M. tuberculosis complex*	51	1.00	0.067	TB
148	Male	68	Positive	Negative	9525503	*M. tuberculosis complex*	519	1.00	0.251	TB
149	Male	39	Positive	Negative	30120257	*M. tuberculosis complex*	1499	1.00	1.510	TB
150	Male	52	Positive	Positive	7700573	*M. tuberculosis complex*	82	1.00	0.100	TB
151	Male	68	Positive	Positive	23059618	*M. tuberculosis complex*	512	1.02	1.060	TB
152	Male	53	Positive	Positive	49249217	*M. tuberculosis complex*	62	1.00	0.090	TB
153	Male	23	Positive	Positive	41442706	*M. tuberculosis complex*	8	1.07	0.020	TB
154	Male	71	Positive	Positive	11033510	*M. tuberculosis complex*	10	1.00	0.010	TB
155	Male	50	Positive	Negative	30179017	*M. tuberculosis complex*	46	1.04	0.060	TB
156	Male	59	Positive	Positive	49227458	*M. tuberculosis complex*	209	1.02	0.290	TB
157	Male	58	Positive	Positive	27439189	*M. tuberculosis complex*	62	1.01	0.090	TB
158	Male	47	Positive	Positive	40306894	*M. tuberculosis complex*	4	1.05	0.010	TB
159	Male	38	Positive	Negative	23765085	*M. tuberculosis complex*	4	1.00	0.005	TB
160	Male	34	Positive	Positive	21296301	*M. tuberculosis complex*	422	1.00	0.515	TB
161	Male	49	Positive	Positive	15807637	*M. tuberculosis complex*	21	1.10	0.019	TB
162	Male	51	Positive	Positive	16773323	*M. tuberculosis complex*	1	1.00	0.002	TB

### Pathogen composition

**Fig 1:**
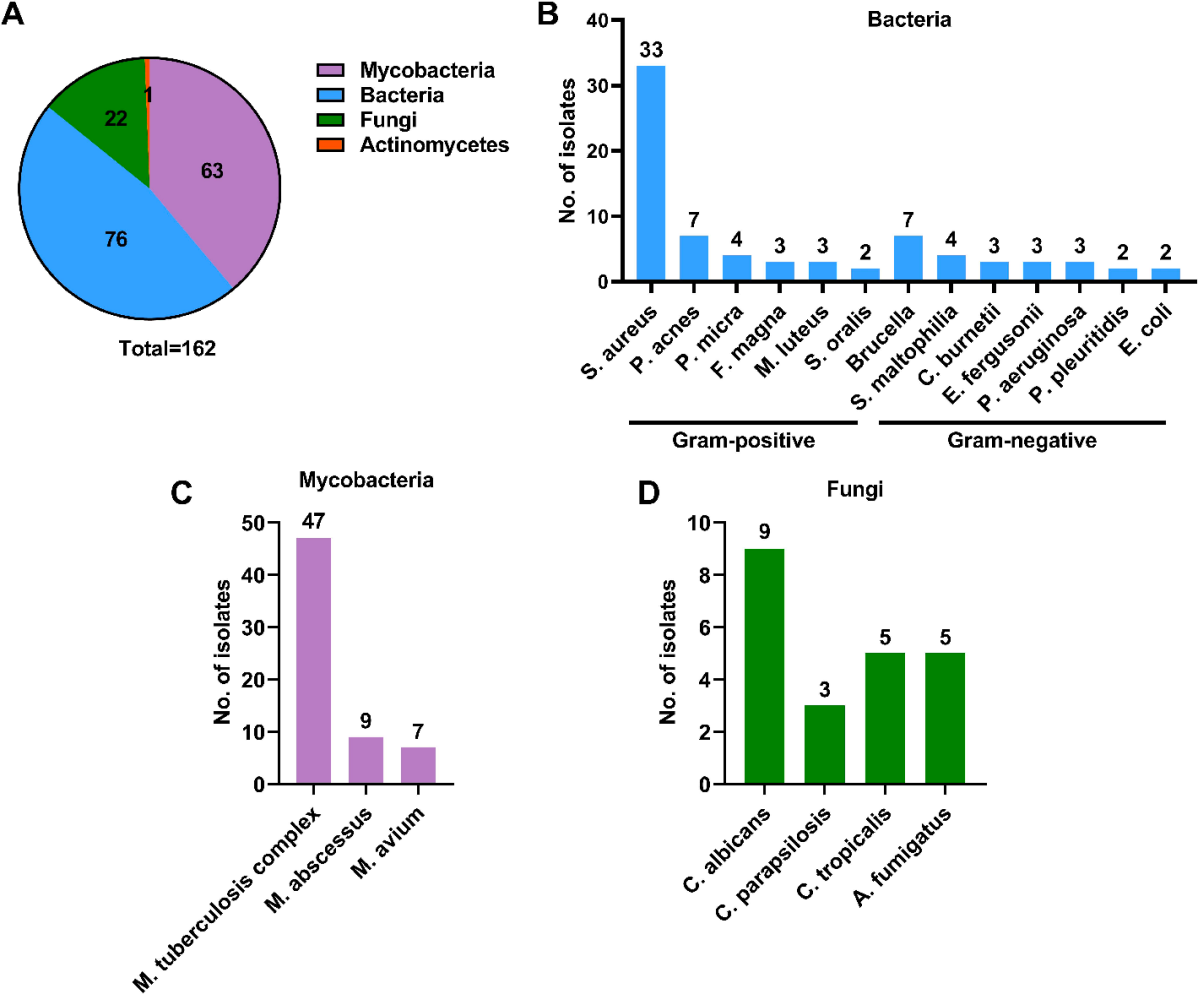
Detection of pathogens by mNGS in specimens obtained from enrolled 162 patients in this study. (**A**)Composition of pathogens stratified to bacteria, mycobacteria, fungi, and actinomycetes. The number shown represents the number of cases infected with the corresponding pathogen; (**B**)composition of bacterial species in the enrolled patients; (**C**)composition of mycobacterial species in the enrolled patients; (**D**)composition of actinomycetal species in the enrolled patients.

As shown in Fig. 1, the mNGS assay could detect potential osteoarticular infection-associated pathogens in all cases, including 76 cases (46.9%) with bacterial, 63 cases (38.9%) with mycobacterial, 22 cases (13.6%) with fungal, and 1 case (0.6%) with actinomycetal species. Sequence read numbers obtained for each pathogen ranged from 1 to 1.3 × 10^6^ reads, with an average of (0.2 ±  1.1) × 10^5^ reads (Table 2). These 162 pathogens were classified into 21 species. The most frequent species detected was *M. tuberculosis complex* (47/162, 29.0%), followed by *S. aureus* (33/162, 20.4%), *M. abscessus* (9/162, 5.6%), and *C. albicans* (9/162, 5.6%; Fig. 1).

### Comparison of the diagnostic performance between mNGS and MGIT culture or Xpert

Taking the “gold standard” TB diagnosis as the standard, among the 162 patients included, the PPV of mNGS, Xpert, and MGIT culture detection was both 100.00%. The NPV of mNGS, Xpert, and MGIT culture and assays were 94.26% (115/122), 98.29% (115/117), and 88.46% (115/130), respectively. The sensitivities of mNGS detection, Xpert, and MGIT culture were 85.11% (40/47), 95.74% (45/47), and 68.08% (32/47), respectively. Therefore, the sensitivity of mNGS detection was similar to that of Xpert and higher than that of MGIT culture. The specificities of mNGS detection, Xpert, and MGIT culture were all 100.00% (Table 3).

**TABLE 3:** Diagnostic performance of mNGS, MGIT culture, and Xpert assay in 162 suspected TB patients

Methods	Sensitivity (%, *n*, 95%CI)	Specificity (%, *n*, 95%CI)	PPV (%, *n*, 95%CI)	NPV (%, *n*, 95%CI)	*P* value (sensitivity)	*P* value (specificity)
mNGS	85.11	100.00	100.00	94.26		
	(40/47	(115/115)	(40/40)	(115/122)		
	(72.9–94.4)	(96.8–100.0)	(91.4–100.0)	(88.9–97.9)		
Xpert	95.74	100.00	100.00	98.29	χ^2^ = 1.12	
	(45/47)	(115/115)	(45/45)	(115/117)	*P* = 0.289^*a*^	*P* = 1.000^*a*^
	(83.9–99.8)	(96.8–100.0)	(92.1–100.0)	(93.2–99.9)		
MGIT culture	68.08	100.00	100.00	88.46	χ2 = 3.368	
	(32/47)	(115/115)	(32/32)	(115/130)	*P* = 0.064^*a*^	*P* = 1.000^*a*^
	(51.5–79.1)	(96.8–100.0)	(89.1–100.0)	(81.0–92.8)		

^*a*^ statistical comparison with mNGS.

### Comparison of the diagnostic effectiveness of mNGS, Xpert, or MGIT culture for TB

**Fig 2:**
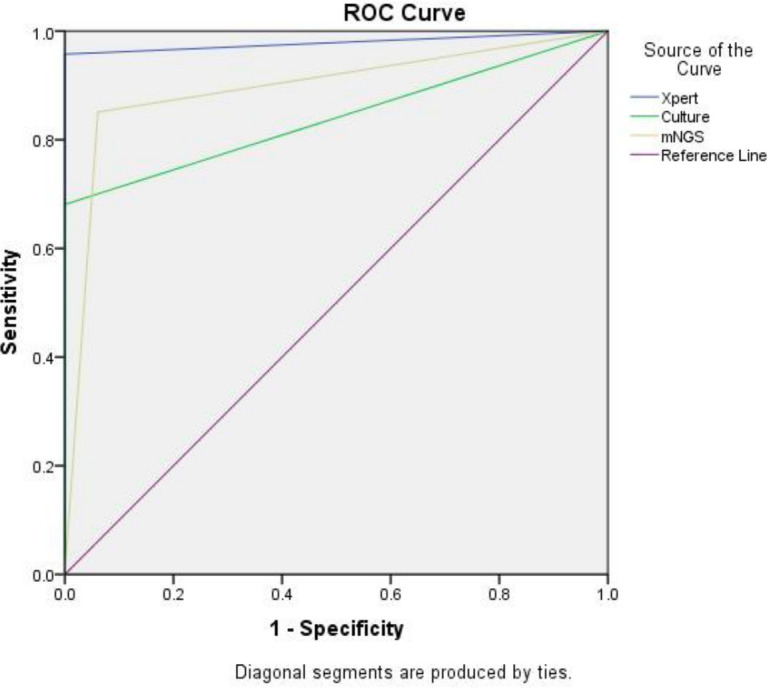
The diagnostic accuracy of diﬀerent detection techniques.

Furthermore, the area under the curve (AUC) value of the mNGS assay was 0.895 (95% CI: 0.830, 0.960), which was greater than that the MGIT culture-based assay of 0.840 (95% CI: 0.757, 0.924), which was similar to 0.979 (95% CI: 0.945, 1.000) for Xpert assay (Fig. 2). Therefore, the above results indicated that the mNGS test has higher diagnostic accuracy in diagnosing suspected osteoarticular TB cases in comparison to MGIT culture and Xpert assays.

## DISCUSSION

Diagnosing osteoarticular TB generally relies on detection of bone morphological changes. However, this approach provides low diagnostic sensitivity for detection of osteoarticular TB infections, since bone lesions are only present in 2% of all TB cases and in only 10%–20% of extrapulmonary TB cases (17, 18). Alternatively, testing of abscess specimens may increase diagnostic sensitivity, although numerous killed bacteria and dead neutrophils within abscess tissue may reduce diagnostic sensitivity for detecting the few surviving pathogenic organisms remaining in such specimens (6). In fact, low pathogen viability within abscesses leads to reduced live pathogen cell recovery rates that render abscess specimens unsuitable for testing via culture-based methods for use in diagnosing osteoarticular TB infections (19).

In spite of nonspecific clinical features and histopathological findings related to mycobacterial infections, diagnosing such infections has mainly relied on pathologic examination findings and mycobacterial culture-based test results. Traditional mycobacterial culture methods, which assess bacterial growth and biochemical characteristics, usually take several weeks to complete. In recent years, molecular biological methods such as NGS have gradually been adopted for mycobacterial identification. For TB diagnosis, NGS has a number of advantages, including its potential to provide both high sensitivity and high specificity through proper selection of reference genomic sequences and, critically, through selection of an appropriate sequence alignment method (20). Clinical guidelines based on expert consensus with regard to metagenomic NGS best practices recommend that in cases lacking clear TB diagnoses (as based on conventional laboratory test results) and successful anti-TB treatment outcomes (within 3 days of treatment completion), specimens should be collected and tested via NGS. Importantly, Pang’s results suggest that mNGS holds great promise as an effective and rapid method for identifying pathogens within abscess specimens that would enable clinicians to administer more effective anti-TB treatments earlier to reduce patient mortality and improve treatment outcomes (19).

In our study, pathogens detected in 83.33% (45/54) of abscess specimens obtained from patients with suspected osteoarticular TB diagnoses were identified as bacteria, thus demonstrating that reliance on clinical findings alone without definitive pathogen test results can lead to misdiagnoses. Furthermore, results of this study revealed that 48.89% (22/45) of bacterial species in abscess specimens were mycobacterial species. Although 86.36% (19/22) abscess samples harbored MTB, around 13.64% (3/22) of abscesses harbored non-tuberculous mycobacterial species that require different therapeutic regimens for elimination than those used to eliminate MTB. Moreover, various fungal species were identified in abscess specimens from other patients that would require clinicians to conduct differential diagnosis in order to administer effective treatments to these patients.

Notably, we also tested and compared diagnostic performance indicators of mNGS, MGIT culture, and Xpert assays. Our results revealed that the sensitivity of mNGS detection was similar to that of Xpert and higher than that of MGIT culture, while the mNGS specificity rates were similar. For diagnosing suspected TB cases, mNGS provided good PPV and NPV rates, in which the PPV rate of mNGS detection was 100% and the mNGS NPV rate was slightly lower than the Xpert rate while was higher than MGIT culture. Meanwhile, the AUC value of the mNGS assay was greater than that the MGIT culture-based assay and was similar to Xpert assay. Taken together, the above results suggest that the use of mNGS for pathogen diagnosis would facilitate the diagnosis of pathogens that are difficult to detect in clinical practice, and a higher pathogen detection rate could be provided by mNGS compared with traditional culture-based test methods (20).

However, some limitations of our study remain: first, this study retrospectively analyzed data on osteoarticular infections in patients hospitalized in a specialized TB hospital, and sampling bias resulting from the retrospective study design may affect the conclusions drawn. Second, interpretation of specimen test results can be confounded by nucleic acid contamination, especially when low levels of microbial biomass were present in the specimen (15). Although NTC was utilized throughout the mNGS workflow, contamination that occurs during sample collection, processing, and testing may lead to false positive results. Therefore, clinicians should be cautious when interpreting the results of mNGS alone and mNGS should be combined with other clinical tests to enable comprehensive judgment.

## Data Availability

The raw sequence reads data set generated for this study can be found in the BioProject database, https://www.ncbi.nlm.nih.gov/bioproject/?term=PRJNA1088114. The data sets used or analyzed during the current study are available from the corresponding author upon reasonable request.
